# Prevalence and Age-Related Patterns in Health Information–Seeking Behaviors and Technology Use Among Skin Cancer Survivors: Survey Study

**DOI:** 10.2196/36256

**Published:** 2022-04-22

**Authors:** Michael Armando Marchetti, Liliane Sar-Graycar, Stephen W Dusza, Japbani K Nanda, Nicholas Kurtansky, Veronica M Rotemberg, Jennifer L Hay

**Affiliations:** Memorial Sloan Kettering Cancer Center, New York, NY, United States

**Keywords:** skin cancer, melanoma, health information, HINTS, internet, cancer, dermatology, information, oncology, survey, analysis

## Abstract

**Background:**

Information is an unmet need among cancer survivors. There is a paucity of population-based data examining the health information–seeking behaviors and attitudes of skin cancer survivors.

**Objective:**

We aimed to identify the prevalence and patterns of health information–seeking behaviors and attitudes among skin cancer survivors across age groups.

**Methods:**

We analyzed population-based data from the 2019 Health Information National Trends Survey 5 (Cycle 3).

**Results:**

The 5438 respondents included 346 (6.4%) skin cancer survivors (mean age 65.8 years); of the 346 skin cancer survivors, the majority were White (96.4% [weighted percentages]), and 171 (47.8%) were men. Most reported having ever looked for health- (86.1%) or cancer-related (76.5%) information; 28.2% stated their last search took a lot of effort, and 21.6% were frustrated. The internet was most often cited as being the first source that was recently used for health or medical information (45.6%). Compared to skin cancer survivors younger than 65 years old, those 65 years of age or older were more likely to see a doctor first for important health information (≥65 years: 68.3%;<65 years: 36.2%; *P*<.001) and less likely to have health and wellness apps (≥65 years: 26.4%; <65 years: 54.0%, *P*=.10), to have watched a health-related YouTube video (≥65 years: 13.3%; <65 years: 27.4%; *P*=.02), and to have used electronic means to look for information (≥65 years: 61.4%;<65 years: 82.3%, *P*<.001)

**Conclusions:**

Searches for health information are common among skin cancer survivors, but behaviors and attitudes are associated with age, which highlights the importance of access to doctors and personalized information sources.

## Introduction

Information is an unmet need that is frequently reported by skin cancer survivors and patients [1–9]. An improved understanding of the etiology and risk factors of a disease and its prevention strategies could facilitate coping mechanisms and improve health care outcomes [10–12]. Most [13], but not all [14], individuals desire as much information as possible about their disease and treatment [3]. A population-based survey [15] in 2003 found that approximately two-thirds of all cancer survivors sought cancer information and the predictors of information-seeking behaviors were younger age, female gender, higher income, and having a regular health care provider.

Few studies have examined information-seeking behaviors, attitudes, and preferences in skin cancer survivors. Survey studies of patients and survivors melanoma have found that many individuals desired more information about their diagnosis, prognosis, or treatment options [4–6,9], expressed high levels of dissatisfaction with the information they received [5,6], and used the internet as a supplementary information resource to their physician [3,6,16]. Internet use and smartphone ownership are strongly associated with age [17,18], however, and melanoma websites have been reported to have poor readability and variable quality [16,19–23]. Patients and survivors of keratinocyte carcinoma who reported dissatisfaction with the information provided have been found to exhibit lower levels of health-related quality of life, increased worry [7], and increased skin cancer–specific and general distress [24,25]. Despite the differences in prognosis between keratinocyte carcinoma and melanoma, the needs, concerns, and levels of distress about diagnosis and need for follow-up appear to be similar in these individuals [8]. There has been recent interest in the development of smartphone and other mobile apps to provide information and education about skin cancer etiology and risk factors, ultraviolet radiation exposure advice, and skin self-examination, as well as tools for analysis or monitoring and tracking of skin lesions [26]. However, the overwhelming majority (99%) of survivors of melanoma have reported never having used a skin cancer–related app [27].

The purpose of this study was to describe and identify age-related factors associated with (1) health information–seeking behaviors and attitudes and (2) use and ownership of technology among a nationally representative sample of skin cancer survivors in the United States. The resultant data would be expected to aid the design of interventions to improve health care outcomes in this patient population.

## Methods

### Ethics

The study was exempt from institutional review board review under US federal regulation [28] because the data were publicly available.

### Study Population, Design, and Setting

Data for this study were obtained from the 2019 Health Information National Trends Survey (HINTS) 5, Cycle 3 [29], which is a nationally representative survey of civilian, noninstitutionalized US adults 18 years or older that was administered by the National Cancer Institute. A detailed description of survey methodology has been published [29]. The sample frame was a random sample of nonvacant residential addresses in the Marketing Systems Group database and was grouped into strata having high or low concentrations of minority populations using census tract–level characteristics from the 2013–2017 American Community Survey data file. An equal-probability sample of addresses was selected from each sampling stratum but oversampling of the high-minority stratum was performed. The total number of addresses selected was 23,430; of these, 6690 (28.6%) were from low minority areas and 16,740 (71.4%) were from high-minority areas. One adult per sampled household was selected to participate in the survey using the next birthday method; a US $2 prepaid monetary incentive was used to encourage participation. The address sample was divided into 3 subsamples: traditional data collection sample using paper-mail survey (n=14,730), web option, offering respondents a choice between responding via paper (English or Spanish) or web (in English only) (n=4350), and web bonus, offering respondents a choice between responding via paper (English or Spanish) or web (in English only), with an additional US $10 incentive for those responding via web (n=4350). The overall response rate for the 23,430 samples was 30.3% (paper-mail 30.2%, web option 29.6%, web bonus 31.5%) [29].

### Study Variables and Statistical Analysis

Descriptive statistics and graphical methods were used to assess the distributions of study variables. The analytic goal was to assess the prevalence and relationships between respondent age and survey responses related to health information–seeking behaviors, attitudes toward health information–seeking, and ownership and use of technology (Multimedia Appendix 1). Respondent age was dichotomized (<65 years or ≥65 years). This age cut-off corresponds to the median age at diagnosis of melanoma [30] and the age for Medicare eligibility [31] in the United States. In addition to these primary independent variables, we also assessed the associations between respondent age and demographic variables, which included sex (male or female), highest grade or level of schooling completed (less than high school, high school, some college, or college), health care coverage (yes or no), respondent race (White, Black, multiple races), total household income (<$50,000 or ≥$50,000), and ability to speak English (very well, well, and not well).

Since the HINTS study has a complex survey design, we utilized jackknife replication weights to adjust standard error estimates. Chi-square statistics along with the weighted relative proportions were used to assess bivariate associations between age and demographic and health information–seeking survey responses. Logistic regression was used to examine the association between respondent age and health information–seeking variables while controlling for respondent demographic characteristics. As there were several modes of survey administration, we evaluated the distribution of demographic characteristics and selected survey responses by mode. Differences in the distribution of these variables by survey mode were assessed by linear regression and chi-square analysis using jackknife estimates (Table S1 in Multimedia Appendix 1). Data management and analysis were completed using StataMP software (version 16.1; StataCorp LLC). Analyses were conducted from March 2020 through January 2021.

## Results

The 5438 HINTS respondents included 346 (6.4%) with self-reported history of any skin cancer (melanoma: n=59; nonmelanoma skin cancer: n=258; both melanoma and nonmelanoma skin cancer: n=29), with a mean age of 65.8 years. Of the 346 respondents with a history of skin cancer, the majority were White (96.4%[weighted percentages]), and 171 were men (47.8%) (Table 1; Table S2 in Multimedia Appendix 1).

### Health Information–Seeking Behaviors

Overall, 86.1% of skin cancer survivors reported having ever looked for information about health or medical topics from any source and 76.5% reported having ever looked for information about cancer from any source (Table 2; Table S3 in Multimedia Appendix 1). During the most recent search for health or medical information, 55.3% reported looking for information for themselves, 11.9% reported looking for information for someone else, and 17.7% reported looking for information for both themselves and someone else. Respondents reported that the internet was the most recent source of health information (45.6%), followed by a doctor or health care provider (20.9%) or other sources (9.5%); 21.6% of respondents felt frustrated in their search for information, and 28.2% of respondents felt it took a lot of effort to get the information they needed.

Compared to survivors aged 65 years and older, survivors <65 years old were more likely to use the internet as their first source of information during their most recent search for information about health or medical topics (<65 years: 59.2%; ≥65 years: 35.2%; *P*=.047) and to have ever looked for information about cancer (<65 years: 86.0%; ≥65 years: 69.2%; *P*=.02). In the model adjusted for sex, income, and English-speaking ability, having ever looked for information about cancer was not associated with being 65 years and older (odds ratio [OR] 1.14, 95% CI 0.81–1.61; *P*=.44) and using the internet as the first source of information about health or medical topics was not associated with being 65 years and older (OR 0.40, 95% CI 0.08–2.08; *P*=.27) (Table 3). No other relationships between general health information–seeking behavior and age were identified.

### Attitudes Toward Health Information–Seeking

A majority of skin cancer survivors (54.4%) reported that they would first go to their doctor if they had a strong need to get information about health or medical topics, followed by the internet (36.9%) or other sources (3.7%). Most respondents reported high levels of confidence in their ability to get advice or information about health or medical topics if needed, with 66.8% completely or very confident, 26.6% somewhat confident, and 4.4% a little confident or not at all confident. A plurality of skin cancer survivors reported a lot of trust in health information from a doctor (79.3%) but not in health information from government health agencies (16.9%), charitable organizations (1.0%), or religious organizations and leaders (2.9%) (*P*<.001 for all comparisons).

Compared to survivors aged 65 years and older, survivors <65 years old were less likely to first go to their doctor for health information (<65 years: 36.2%; ≥65 years: 68.3%; *P*<.001) and more likely to go to the internet (<65 years: 55.3%; ≥65 years: 22.9%; *P*<.001). In the model adjusted for sex, income, and English-speaking ability, going to their doctor first was strongly associated with being 65 years and older (OR 3.88, 95% CI 1.82–8.23; *P*=.001) compared to going to the internet first. No other relationships between general health information–seeking behavior and age were identified.

### Ownership and Use of Technology

Although 81.0% of skin cancer survivors reported owning a smartphone or tablet device, 13.2% owned a basic mobile phone only. In the past 12 months, 68.2% of survivors had used a smartphone, computer, or other electronic means to look for health or medical information pertinent to their health, and 26.2% had used the internet to look for information about cancer. A minority of survivors reported having watched a health-related video on YouTube in the past 12 months (19.4%) or having apps related to health and wellness on a tablet or smartphone (38.3%).

Age was strongly associated with the ownership and use of technology (Table 2). Compared to survivors aged 65 years and older, survivors younger than 65 years old were more likely to have apps related to health and wellness (<65 years: 54.0%; ≥65 years: 26.4%; *P*=.10), more likely to have watched a health-related YouTube video (<65 years: 27.4%; ≥65 years: 13.3%; *P*=.02), and more likely to have used an electronic means to look for health and medical information (<65 years: 82.3%; ≥65 years: 61.4%; *P*<.001). After adjusting for sex, income, and English-speaking ability, having health and wellness apps (OR 0.35, 95% CI 0.13–0.93; *P*=.04), watching a health-related YouTube video (OR 0.38, 95% CI 0.17–0.84; *P*=.02), and using electronic means to look for health information (OR 0.17, 95% CI 0.05–0.56; *P*=.004) were associated with being <65 years old (Table 3).

## Discussion

### General

We found that health- and cancer-related information-seeking behaviors are common among skin cancer survivors but that 21.6% of respondents felt frustrated, and 28.2% felt that their most recent search for health information took significant effort. Age was strongly associated with survivor preferences and use of technology. Younger survivors were more likely to use and prefer technology-based means, such as the internet, health and wellness apps, or YouTube, to access information. These findings are relevant to clinical practice as well as to research efforts aimed at improving patient education and primary and secondary prevention behaviors, particularly as the population older than 65 years is rapidly expanding in the United States [32] and technology ownership and use varies by age [17,18,33].

Previous studies [3,34] have tended to survey survivors in tertiary-care specialty clinics, limiting generalizability. Brutting et al [3] found that the internet was strongly preferred as a media information resource by younger (<55 years) more than older patients with or who had a history of melanoma in Germany and that the information source most frequently used by patients with or who had a history of melanoma was their physician, followed by family or friends, other health care professionals, the internet, and booklets. Self-help groups, cancer counseling centers, and health insurance companies were infrequently used as an information resource [3]. Damude et al [34] conducted a prospective study in which, prior to an outpatient visit, a printed melanoma brochure and links to 2 educational YouTube videos about skin self-examination were sent to Dutch stage I-II melanoma survivors, who subsequently reported that they preferred their treating physician over YouTube videos or printed brochures as the primary information source. Their findings [34] and ours highlight that skin cancer survivors strongly value their doctor as an information resource. In addition, the majority of melanoma survivors felt that YouTube videos gave complementary information, had additional value and increased their confidence; most would recommend them to other patients [34]. Interestingly, in our study, we found very low use of health-related videos from YouTube, which suggests that although there may possibly be interest in this medium among skin cancer survivors, they may not be aware of, or know how to, identify or access, reputable resources. This challenge was highlighted by Petukhova et al [35], who found that 87% of posts involving medical advice shared in Facebook support groups for keratinocyte carcinoma survivors included unsupported claims.

Web-based and print-based materials for melanoma education have been recently complemented by device (such as smartphone) apps, with which people interact with daily. With the rise in smartphone ownership, apps are a promising resource to help encourage patients and survivors to increase preventive health behaviors, including ultraviolet radiation protective behaviors and skin self-examinations [36–52]. However, because smartphone ownership varies by demographics [18], alternative strategies must be developed in parallel to prevent health care disparities.

Interestingly, few respondents reported high levels of trust in information about health or medical topics from government health agencies. This is consistent with the findings of a 2021 survey [53] of 1305 US adults that showed that the American public has significantly higher trust in health care professionals than in public health institutions and agencies. Addressing concerns of a lack of trust in US public health institutions and agencies, therefore, appears to be an opportunity for improvement. This is particularly relevant for skin cancer as it is the most commonly diagnosed cancer in the United States, most cases are preventable, and it was the topic of the US surgeon general’s 2014 call to action [54].

### Limitations

Data were limited by the survey response rate and the potential for recall and selection biases. Additionally, these data were in relation to general health information and not specific to skin cancer–related information. Given the low number of non-White skin cancer survivors in the data set, our findings may not be generalizable to other races and ethnicities. Selection bias was limited by the use of data from a rigorously conducted, population-based, nationally representative sample, that provided modest monetary compensation. Finally, we did not analyze data from prior HINTS surveys to determine temporal changes in measures, and we did not assess similarities or differences in information-seeking behaviors and use of technology between skin cancer survivors and other individuals.

### Conclusion

Searches for health information are common among skin cancer survivors. Although behaviors and attitudes are associated with age, individuals of all ages have varied preferences, highlighting the importance of access to doctors and personalized information sources.

## Supplementary Material

Multimedia Appendix 1

## Figures and Tables

**Table 1.** Demographic characteristics of skin cancer respondents stratified by age (<65 years vs ≥65 years).

Variable	Respondents, n (weighted %)	Respondents by age		*P* value
		<65 years, n (weighted %)	≥65 years, n (weighted %)	

**Sex**				.14
Missing	23 (5.1)	7 (3.5)	16 (6.4)	
Male	171 (47.8)	43 (40.4)	128 (53.5)	
Female	152 (47.0)	63 (56.1)	89 (40.1)	
**Education**				.08
Missing	2 (0.1)	0 (0)	2 (0.2)	
Less than high school	9 (3.2)	1 (0.5)	8 (5.3)	
High school	51 (22.1)	11 (16.1)	40 (26.6)	
Some college	102 (39.8)	29 (41.0)	73 (38.9)	
College	182 (34.8)	72 (42.5)	110 (28.9)	
**Health insurance**				.21
Missing	4 (1.3)	0 (0)	4 (2.3)	
Yes	339 (97.7)	112 (99.7)	227 (96.1)	
No	3 (1.0)	1 (0.3)	2 (1.5)	
**Race**				.003
Missing	14 (3.1)	2 (0.7)	12 (5.0)	
White	326 (96.4)	107 (98.4)	219 (94.9)	
Black	1 (0)	1 (0.1)	0 (0)	
Multiple	5 (0.4)	3 (0.9)	2 (0.1)	
**Income**				.23
Missing	34 (10.9)	6 (7.6)	28 (13.4)	
<$50,000	141 (45.2)	41 (41.1)	100 (48.4)	
≥$50,000	171 (43.9)	66 (51.4)	105 (38.2)	
**Speaks English**				.02
Missing	2 (0.5)	1 (0.6)	1 (0.4)	
Very well	318 (92.7)	109 (96.9)	209 (89.5)	
Well	21 (5.7)	1 (11)	20 (9.3)	
Not well	5 (1.1)	2 (1.5)	3 (0.7)	

**Table 2.** Distribution of health seeking behaviors by skin cancer respondents stratified by age (<65 years vs ≥65 years).

Variable	Respondents (n=346)	Respondents by age		*P* value
	n (weighted %)	<65 years (n=113), n (weighted %)	≥65 years (n=233), n (weighted %)	

**Health information–seeking behaviors**				
**Have you ever looked for information about health or medical topics from any source?**				.10
Missing	7 (1.5)	2 (1.0)	5 (1.8)	
Yes	304 (86.1)	103 (91.9)	201 (81.7)	
No	35 (12.4)	8 (7.1)	27 (16.5)	
**Have you ever looked for information about cancer from any source?**				.02
Missing	6 (1.6)	2 (1.9)	4 (1.4)	
Yes	262 (76.5)	93 (86.0)	169 (69.2)	
No	78 (21.9)	18 (12.1)	60 (29.4)	
**The most recent time you looked for information about health or medical topics, who was it for?**				.84
Missing	45 (15.1)	12 (10.2)	33 (19.0)	
Myself	187 (55.3)	60 (60.1)	127 (51.5)	
Someone else	47 (11.9)	16 (12.5)	31 (11.5)	
Both myself and someone else	67 (17.7)	25 (17.3)	42 (18.0)	
**The most recent time you looked for information about health or medical topics, where did you go first?**				.047
Missing	77 (24.0)	23 (18.6)	54 (28.1)	
Doctor	71 (20.9)	13 (14.7)	58 (25.5)	
Internet	168 (45.6)	69 (59.2)	99 (35.2)	
Other	30 (9.5)	8 (7.5)	22 (11.1)	
**You felt frustrated during your search for the information.**				.88
Missing	62 (18.6)	13 (10.3)	49 (24.9)	
Strongly agree	25 (6.6)	9 (5.7)	16 (7.3)	
Somewhat agree	59 (15.0)	20 (16.5)	39 (13.9)	
Somewhat disagree	76 (20.7)	26 (22.6)	50 (19.3)	
Strongly disagree	124 (39.1)	45 (45.0)	79 (34.6)	
**It took a lot of effort to get the information you needed.**				.21
Missing	47 (15.6)	12 (10.2)	35 (19.7)	
Strongly agree	30 (7.7)	11 (6.7)	19 (8.6)	
Somewhat agree	82 (20.5)	19 (17.1)	63 (23.1)	
Somewhat disagree	87 (23.4)	29 (22.6)	58 (24.0)	
Strongly disagree	100 (32.8)	42 (43.5)	58 (24.7)	
**Attitudes toward health information–seeking**				
**Imagine that you had a strong need to get information about health or medical topics. Where would you go first?**				<.001
Missing	17 (5.1)	7 (6.0)	10 (4.4)	
Doctor or health care	179 (54.4)	39 (36.2)	140 (68.3)	
Internet	128 (36.9)	60 (55.3)	68 (22.9)	
Other	22 (3.7)	7 (2.6)	15 (4.5)	
**Overall, how confident are you that you could get advice or information about health or medical topics if you needed it?**				.80
Missing	10 (2.3)	3 (1.1)	7 (3.2)	
Completely confident	90 (29.4)	32 (28.4)	58 (30.2)	
Very confident	133 (37.4)	41 (39.0)	92 (36.2)	
Somewhat confident	94 (26.6)	28 (25.5)	66 (27.4)	
A little confident	10 (3.0)	6 (4.61)	4 (1.7)	
Not confident at all	9 (1.4)	3 (1.4)	6 (1.3)	
**In general, how much would you trust information about health or medical topics from each of the following?**				
**A doctor**				.52
Missing	7 (1.4)	2 (1.0)	5 (1.8)	
A lot	261 (79.3)	84 (81.8)	177 (77.4)	
Some-Not at all	78 (19.2)	27 (17.3)	51 (20.8)	
**Family or friends**				.82
Missing	17 (5.1)	3 (2.3)	14 (7.3)	
A lot	20 (6.8)	9 (7.5)	11 (6.3)	
Some-Not at all	309 (88.0)	101 (90.1)	208 (86.4)	
**Government health agencies**				.60
Missing	21 (5.0)	2 (1.0)	19 (8.2)	
A lot	59 (16.9)	25 (16.0)	34 (17.6)	
Some-Not at all	266 (78.0)	86 (83.0)	180 (74.2)	
**Charitable organizations**				.30
Missing	21 (5.3)	2 (1.0)	19 (8.6)	
A lot	5 (1.0)	3 (1.5)	2 (0.5)	
Some-Not at all	320 (93.8)	108 (97.5)	212 (90.9)	
**Religious organizations and leaders**				.57
Missing	20 (4.3)	2 (1.0)	18 (6.9)	
A lot	4 (2.9)	2 (4.1)	2 (2.0)	
Some-Not at all	322 (92.8)	109 (94.9)	213(91.2)	
**Ownership and technology use**				
**Please indicate if you have each of the following.**				.045
Missing	5 (1.6)	1 (1.5)	4 (1.6)	
Tablet computer	19 (8.5)	5 (7.8)	14 (9.0)	
Smartphone	99 (31.0)	35 (33.7)	64 (29.0)	
Basic cell phone only	45 (13.2)	6 (5.7)	39 (18.9)	
None	22 (4.7)	2 (1.1)	20 (7.5)	
Multiple devices selected	156 (41.1)	64 (50.2)	92 (34.1)	
**On your tablet or smartphone, do you have any apps related to health and wellness?**				.10
Missing	75 (19.7)	10 (8.4)	65 (28.3)	
Yes	138 (38.3)	66 (54.0)	72 (26.4)	
No	113 (30.2)	33 (30.2)	80 (30.2)	
Don’t know	20 (11.8)	4 (7.4)	16 (15.1)	
**In the past 12 months, have you used the internet to look for information about cancer for yourself?**				.86
Missing	66 (23.8)	10 (11.5)	56 (33.2)	
Yes	93 (26.2)	38 (31.1)	55 (22.4)	
No	187 (50.0)	65 (57.4)	122 (44.4)	
**In the last 12 months, have you used the internet to watch a health-related video on YouTube?**				.02
Missing	6 (0.9)	0 (0)	6 (1.6)	
Yes	77 (19.4)	38 (27.4)	39 (13.3)	
No	263 (79.7)	75 (72.6)	188 (85.1)	
**In the past 12 months, have you used a computer, smartphone, or other electronic device to look for health or medical information for yourself?**				<.001
Missing	7 (2.0)	2 (1.8)	5 (2.2)	
Yes	236 (68.2)	93 (82.3)	143 (61.4)	
No	103 (29.8)	18 (15.9)	85 (36.5)	

**Table 3.** Associations between age (<65 years vs ≥65 years) and health information–seeking variables for skin cancer survivors.

Variable	Unadjusted odds ratio (95% CI)	*P* value	Adjusted^a^ odds ratio (95% CI)	*P* value

**Have you ever looked for information about health or medical topics from any source?**				
Yes	2.59 (0.7–9.58)	.15	1.09 (0.76–1.57)	.62
No	1.0^b^	—^c^	1.0^b^	—
**The most recent time you looked for information about health or medical topics, who was it for?**				
Myself	0.93 (0.41–2.11)	.86	0.91 (0.37–2.23)	.83
Someone else	1.0^b^	—	1.0^b^	—
Both myself and someone else	1.13 (0.46–2.76)	.79	1.17 (0.42–3.25)	.75
**The most recent time you looked for information about health or medical topics, where did you go first?**				
Doctor	1.18 (0.26–5.30)	.83	1.16 (0.2–5.9)	.86
Internet	0.40 (0.09–1.83)	.23	0.40 (0.08–2.08)	.27
Other	1.0^b^	—	1.0^b^	—
**Have you ever looked for information about cancer from any source?**				
Yes	1.14 (0.95–1.36)	.16	1.14 (0.81–1.61)	.44
No	1.0^b^	—	1.0^b^	—
**Please indicate if you have a tablet, smartphone, cell phone, basic cell phone, none, or multiple devices.**				
Tablet computer	1.7 (0.21–13.94)	.61	1.55 (0.09–26.82)	.76
Smartphone	1.27 (0.65–2.47)	.48	1.44 (0.62–3.36)	.39
Basic cell phone	4.88 (1.24–19.24)	.03	3.64 (0.7–18.95)	.12
None	10.36 (0.82–130.35)	.07	8.13 (0.64–102.8)	.10
Multiple devices	1.0^b^	—	1.0^b^	—
**On your tablet or smartphone, do you have any apps related to health and wellness?**				
Yes	0.42 (0.25–0.70)	0.001	0.35 (0.13–0.93)	.04
No or don’t know	1.0^b^	—	1.0^b^	—
**In the last 12 months, have you used the internet to watch a health-related video on YouTube?**				
Yes	0.42 (0.19–0.88)	0.02	0.38 (0.17–0.84)	.02
No	1.0^b^	—	1.0^b^	—
**In the past 12 months, have you used a computer, smartphone, or other electronic device to get health-related information?**				
Yes	0.23 (0.10–0.53)	.001	0.17 (0.05–0.56)	.004
No	1.0^b^	—	1.0^b^	—
**Imagine that you had a strong need to get information about health or medical topics. Where would you go first?**				
Internet	1.0^b^	—	1.0^b^	—
Doctor	4.56 (2.01–10.37)	.001	3.88 (1.82–8.23)	.001
Elsewhere	4.19 (0.9–19.46)	.07	5.24 (0.99–27.84)	.05
**Overall, how confident are you that you could get advice or information about health or medical topics if you needed it?**				
Completely confident	1.0^b^		1.0^b^	—
Very confident	0.87 (0.36–2.14)	.76	0.90 (0.37–2.20)	.82
Somewhat or not confident	0.91 (0.38–2.14)	.82	0.80 (0.33–1.92)	.61
**You felt frustrated during your search for the information**				
Strongly agree or somewhat agree	1.0^b^	—	1.0^b^	—
Somewhat disagree	0.89 (0.33–2.41)	.82	1.08 (0.38–3.04)	.88
Strongly disagree	0.8 (0.38–1.71)	.57	0.99 (0.43–2.26)	.97
**It took a lot of effort to get the information you needed**				
Strongly agree	1.0^b^	—	1.0^b^	—
Somewhat agree	1.05 (0.25–4.32)	.95	1.62 (0.33–8.08)	.55
Somewhat disagree	0.82 (0.19–3.52)	.79	1.69 (0.28–10.35)	.56
Strongly disagree	0.44 (0.12–1.58)	.20	0.64 (0.15–2.68)	.53

a The model was adjusted for respondent sex, income, and English-speaking ability.

b Reference.

c No data or not applicable.

## Abbreviations

HINTS: Health Information National Trends Survey
OR: odds ratio
