# Supplementary material for: Prevalence and Age-Related Patterns in Health Information–Seeking Behaviors and Technology Use Among Skin Cancer Survivors: Survey Study
Source: JMIR Dermatol. 2022 Apr 22;5(2):e36256. doi: 10.2196/36256 (PMC9910806; doi:10.2196/36256)
Supplement: Multimedia Appendix 1 [file derma_v5i2e36256_app1.docx]

**Online Supplement**

**eMethods**

A list of the measures used in analyses is below. A complete list of all questions in the HINTS survey instrument is available online.^1^

**Section A.**

**A1.** Have you ever looked for information about health or medical topics from any source? (yes/no). If yes -> A2, A3, A4.

**A2**. The most recent time you looked for information about health or medical topics, where did you go first? Mark only one. (books / brochures, pamphlets, etc. / cancer organization / family / friend, co-worker / doctor or health care provider / internet / library / magazines / newspapers / telephone information number / complementary, alternative, or unconventional practitioner).

**A3**. The most recent time you looked for information about health or medical topics, who was it for? (myself, someone else, both myself and someone else)

**A4.** Based on the results of your most recent search for information about health or medical topics, how much do you agree or disagree with each of the following statements

**A4a.** It took a lot of effort to get the information you needed. (strongly agree / somewhat agree / somewhat disagree / strongly disagree)

**A4b.** You felt frustrated during your search for the information. (strongly agree / somewhat agree / somewhat disagree / strongly disagree)

**A5.** Overall, how confident are you that you could get advice or information about health or medical topics if you needed it? (completely confident / very confident / somewhat confident / a little confident / not confident at all).

**A6.** In general, how much would you trust information about health or medical topics from each of the following?

**A6a.** A doctor (not at all, a little, some, a lot)

**A6b.** Family or friends (not at all, a little, some, a lot)

**A6c.** Government health agencies (not at all, a little, some, a lot)

**A6d.** Charitable organizations (not at all, a little, some, a lot)

**A6e.** Religious organizations and leaders (not at all, a little, some, a lot)

**A7.** Imagine that you had a strong need to get information about health or medical topics. Where would you go first? Mark only one. (brooks / brochures, pamphlets, etc. / cancer organization / family / friend, co-worker / doctor or health care provider / internet / library / magazines / newspapers / telephone information number / complementary, alternative, or unconventional practitioner / other)

**A8.** Have you ever looked for information about cancer from any source? (yes/no)

**Section B.**

**B3.** In the past 12 months, have you used the Internet to look for information about cancer for yourself? (yes/no)

**B6.** Please indicate if you have each of the following. Mark all that apply. (Tablet computer / Smartphone / Basic cell phone only / I do not have any of the above)

**B7.** On your tablet or smartphone, do you have any “apps” related to health and wellness? (yes/no/don’tknow)

**Section C.**

**C7.** Are you currently covered by any of the following types of health insurance or health coverage plans?

**C7a.** Insurance through a current or former employer or union (yes/no)

**C7b.** Insurance purchased directly from an insurance company (yes/no)

**C7c.** Medicare, for people 65 and older, or people with certain disabilities (yes/no)

**C7d.** Medicaid, Medical Assistance, or any kind of government-assistance plan for those with low incomes or a disability (yes/no)

**C7e.** TRICARE or other military health care (yes/no)

**C7f.** VA (yes/no)

**C7g.** Indian Health Service (yes/no)

**C7h.** Any other type of health insurance or health coverage plan (Specify) (yes/no)

**Section O.**

**O1.** What is your age?

**O3.** What is your highest grade or level of schooling you completed? (less than 8 years / 8 through 11 years / 12 years or completed high school / post high school training other than college (vocational or technical) / some college / college graduate / postgraduate)

**O4.** How well do you speak English? (very well / well / not well / not at all)

**O6.** What is your race? One or more categories may be selected. Mark all that apply. (White / Black or African American / American Indian or Alaska native / Asian Indian / Chinese / Filipino / Japanese / Korean / Vietnamese / other Asian / Native Hawaiian / Guamanjan or Chamorro / Samoan / other Pacific Islander)

**O12.** Thinking about members of your family living in this household, what is your combined annual income, meaning the total pre-tax income from all sources earned in the past year? ($0 to $9,999 / $10,000 to 14,999 / $15,000 to $19,999 / $20,000 to $34,999 / $35,000 to $49,999 / $50,000 to $74,999 / $75,000 to $99,999 / $100,000 to $199,999 / $200,000 or more)

eTable 1. Bivariate associations between participant characteristics and mode of delivery of HINTS survey.

| Variable | Coding | Mode of Survey Administration | | | |  | P-value |
| --- | --- | --- | --- | --- | --- | --- | --- |
|  |  | Mail only | Web/Mail | Web/Mail + bonus | Total |  |  |
| Age | Continuous | 65.21 | 67.92 | 65.36 |  |  | 0.526*  0.963** |
|  |  |  |  |  |  |  |  |
| Education | Less than HS | 0.0115 | 0.1191 | 0 | 0.0322 |  | 0.0119 |
|  | HS | 0.2332 | 0.1261 | 0.3351 | 0.2209 |  |  |
|  | Some college | 0.38 | 0.5378 | 0.2321 | 0.3984 |  |  |
|  | College | 0.3752 | 0.217 | 0.4328 | 0.3485 |  |  |
|  |  |  |  |  |  |  |  |
| Sex | Male | 0.5295 | 0.4645 | 0.3907 | 0.5042 |  | 0.5975 |
|  | Female | 0.4705 | 0.5355 | 0.6093 | 0.4958 |  |  |
|  |  |  |  |  |  |  |  |
| Race | Missing | 0.0219 | 0.075 | 0.0087 | 0.0314 |  | 0.3515 |
|  | White | 0.9734 | 0.925 | 0.9784 | 0.9641 |  |  |
|  | Black | 3.7E-04 | 0 | 0 | 2.6E-04 |  |  |
|  | Multiple | 0.0043 | 0 | 0.0128 | 0.0042 |  |  |
|  |  |  |  |  |  |  |  |
| Income | <50k | 0.4496 | 0.6794 | 0.5965 | 0.5074 |  | 0.0601 |
|  | 50k+ | 0.5504 | 0.3206 | 0.4035 | 0.4926 |  |  |
|  |  |  |  |  |  |  |  |
| Trust doctor | Not at all to Some | 0.1996 | 0.2341 | 0.076 | 0.1952 |  | 0.311 |
|  | A lot | 0.8004 | 0.7659 | 0.924 | 0.8048 |  |  |
|  |  |  |  |  |  |  |  |
| A1. Have you ever looked for information about health or medical topics from any source? | Yes | 0.873 | 0.8794 | 0.8697 | 0.874 |  | 0.9937 |
|  | No | 0.127 | 0.1206 | 0.1303 | 0.126 |  |  |
|  |  |  |  |  |  |  |  |
| A7. Imagine that you had a strong need to get information about health or medical topics. Where would you go first? | Doctor | 0.5408 | 0.6294 | 0.6838 | 0.5728 |  | 0.6694 |
|  | Internet | 0.4144 | 0.3472 | 0.2899 | 0.3886 |  |  |
|  | Else | 0.0448 | 0.0235 | 0.0263 | 0.0386 |  |  |
|  |  |  |  |  |  |  |  |
| B6. Please indicate if you have each of the following. | Tablet computer | 0.0349 | 0.2205 | 0.1796 | 0.0863 |  | 0.0088 |
|  | Smartphone | 0.3526 | 0.2905 | 0.0868 | 0.315 |  |  |
|  | Basic cell phone | 0.0987 | 0.2275 | 0.1909 | 0.1336 |  |  |
|  | None | 0.0475 | 0.0184 | 0.116 | 0.048 |  |  |
|  | Multiple devices | 0.4663 | 0.243 | 0.4267 | 0.4171 |  |  |
|  |  |  |  |  |  |  |  |
| A5. Overall, how confident are you that you could get advice or information about health or medical topics if you needed it? | Completely confident | 0.2812 | 0.3693 | 0.2996 | 0.301 |  | 0.7803 |
|  | Very confident | 0.3714 | 0.4032 | 0.4195 | 0.3825 |  |  |
|  | Somewhat confident | 0.2871 | 0.2204 | 0.271 | 0.2719 |  |  |
|  | A little confident | 0.04 | 0.0072 | 0.0099 | 0.0305 |  |  |
|  | Not confident | 0.0202 | 0 | 0 | 0.0141 |  |  |

* p-value from linear regression for the comparison between mail-only and web/mail

** P-value from linear regression for the comparison between mail-only and web/mail+bonus

eTable2 Distribution of demographic characteristics of skin cancer respondents stratified by age (<65 years vs. 65+ years).

| Variable | weighted %  (Std. Error) |  | Respondent Age | | P-value |
| --- | --- | --- | --- | --- | --- |
|  |  |  | <65  weighted %  (Std. Error) | 65+  weighted %  (Std. Error) |  |
| Sex |  |  |  |  |  |
| Missing | 5.14 (0.0158) |  | 3.47 (0.0185) | 6.41 (0.0254) | 0.1377 |
| Male | 47.83 (0.0453) |  | 40.39 (0.0761) | 53.52 (0.0497) |  |
| Female | 47.03 (0.0438) |  | 56.14 (0.078) | 40.08 (0.0479) |  |
|  |  |  |  |  |  |
| Education |  |  |  |  |  |
| Missing | 0.12 (0.00097) |  | 0 (0) | 0.22 (0.0017) | 0.0753 |
| Less than HS | 3.22 (0.0162) |  | 0.46 (0.0047) | 5.33 (0.0284) |  |
| HS | 22.06 (0.0307) |  | 16.1 (0.049) | 26.62 (0.0461) |  |
| Some college | 39.79 (0.0338) |  | 40.97 (0.0619) | 38.89 (0.0555) |  |
| College | 34.81 (0.0318) |  | 42.48 (0.0581) | 28.94 (0.0371) |  |
|  |  |  |  |  |  |
| Health Insurance |  |  |  |  |  |
| Missing | 1.31 (0.0096) |  | 0 (0) | 2.32 (0.0169) | 0.2137 |
| Yes | 97.69 (0.0118) |  | 99.73 (0.0028) | 96.14 (0.0208) |  |
| No | 0.99 (0.0073) |  | 0.27 (0.0028) | 1.54 (0.0129) |  |
|  |  |  |  |  |  |
| Race |  |  |  |  |  |
| Missing | 3.14 (0.0127) |  | 0.71 (0.0052) | 5 (0.0217) | 0.003 |
| White | 96.41 (0.013) |  | 98.37 (0.0084) | 94.91 (0.0217) |  |
| Black | 0.026 (0.00027) |  | 0.061 (0.00062) | 0 (0) |  |
| Multiple | 0.42 (0.0028) |  | 0.86 (0.0065) | 0.092 (0.0007) |  |
|  |  |  |  |  |  |
| Income |  |  |  |  |  |
| Missing | 10.89 (0.0253) |  | 7.57 (0.0364) | 13.43 (0.0321) | 0.2287 |
| <50k | 45.22 (0.0416) |  | 41.07 (0.0748) | 48.38 (0.0474) |  |
| 50k+ | 43.89 (0.0448) |  | 51.36 (0.0733) | 38.19 (0.0469) |  |
|  |  |  |  |  |  |
| Speaks English |  |  |  |  |  |
| Missing | 0.49 (0.0034) |  | 0.57 (0.0058) | 0.43 (0.0043) | 0.0236 |
| Very well | 92.73 (0.0177) |  | 96.91 (0.0153) | 89.53 (0.0305) |  |
| Well | 5.74 (0.0169) |  | 1.06 (0.0107) | 9.31 (0.0291) |  |
| Not well | 1.05 (0.0053) |  | 1.46 (0.0109) | 0.74 (0.0047) |  |

eTable3. Distribution of health seeking behaviors by skin cancer respondents stratified by age (<65 years vs. 65+ years).

| Variable | | Coding |  | Overall |  | Respondent Age | | P-value |
| --- | --- | --- | --- | --- | --- | --- | --- | --- |
|  |  |  |  | weighted % (Std. Error) |  | <65  weighted % (Std. Error) | 65+  weighted % (Std. Error) |  |
|  | |  |  |  |  |  |  |  |
| **Health Information Seeking Behaviors** | | | | | | | | |
| Have you ever looked for information about health or medical topics from any source? | | Missing |  | 1.45 (0.0062) |  | 0.98 (0.0072) | 1.81 (0.0096) | 0.1006 |
|  |  | Yes |  | 86.13 (0.0244) |  | 91.88 (0.0326) | 81.73 (0.0379) |  |
|  |  | No |  | 12.42 (0.0239) |  | 7.14 (0.0321) | 16.46 (0.0376) |  |
|  | |  |  |  |  |  |  |  |
| Have you ever looked for information about cancer from any source? | | Missing |  | 1.61 (0.0101) |  | 1.91 (0.0159) | 1.38 (0.0074) | 0.0156 |
|  |  | Yes |  | 76.5 (0.0288) |  | 86.02 (0.0424) | 69.23 (0.0421) |  |
|  |  | No |  | 21.89 (0.0291) |  | 12.07 (0.0411) | 29.4 (0.0426) |  |
|  | |  |  |  |  |  |  |  |
| The most recent time you looked for information about health or medical topics, who was it for? | | Missing |  | 15.14 (0.0264) |  | 10.15 (0.0334) | 18.95 (0.0379) | 0.8453 |
|  |  | Myself |  | 55.26 (0.0361) |  | 60.14 (0.0535) | 51.54 (0.0529) |  |
|  |  | Someone else |  | 11.92 (0.0231) |  | 12.45 (0.0334) | 11.51 (0.0269) |  |
|  |  | Both myself and someone else |  | 17.68 (0.0283) |  | 17.26 (0.0374) | 18.01 (0.0371) |  |
|  | |  |  |  |  |  |  |  |
| The most recent time you looked for information about health or medical topics, where did you go first? | | Missing |  | 23.99 (0.0332) |  | 18.6 (0.0476) | 28.12 (0.0377) | 0.0471 |
|  |  | Doctor |  | 20.85 (0.0287) |  | 14.7 (0.05) | 25.55 (0.0341) |  |
|  |  | Internet |  | 45.62 (0.0432) |  | 59.2 (0.0692) | 35.25 (0.0394) |  |
|  |  | Other |  | 9.53 (0.0263) |  | 7.5 (0.0323) | 11.08 (0.041) |  |
|  | |  |  |  |  |  |  |  |
| You felt frustrated during your search for the information. | | Missing |  | 18.57 (0.027) |  | 10.3 (0.0334) | 24.88 (0.0402) | 0.8760 |
|  |  | Strongly agree |  | 6.59 (0.0169) |  | 5.71 (0.0242) | 7.27 (0.0237) |  |
|  |  | Somewhat agree |  | 15.03 (0.0257) |  | 16.45 (0.042) | 13.94 (0.0279) |  |
|  |  | Somewhat disagree |  | 20.7 (0.0294) |  | 22.57 (0.0545) | 19.27 (0.0386) |  |
|  |  | Strongly disagree |  | 39.11 (0.0363) |  | 44.97 (0.0621) | 34.63 (0.0423) |  |
|  | |  |  |  |  |  |  |  |
| It took a lot of effort to get the information you needed. | | Missing |  | 15.56 (0.0267) |  | 10.15 (0.0334) | 19.7 (0.0382) | 0.2072 |
|  |  | Strongly agree |  | 7.74 (0.0179) |  | 6.65 (0.0275) | 8.56 (0.0256) |  |
|  |  | Somewhat agree |  | 20.51 (0.0293) |  | 17.11 (0.0458) | 23.1 (0.0364) |  |
|  |  | Somewhat disagree |  | 23.39 (0.03) |  | 22.6 (0.0525) | 23.99 (0.0479) |  |
|  |  | Strongly disagree |  | 32.81 (0.0357) |  | 43.49 (0.0632) | 24.65 (0.0404) |  |
|  | |  |  |  |  |  |  |  |
| **Attitudes Toward Health Information Seeking** | | | | | | | | |
| Imagine that you had a strong need to get information about health or medical topics. Where would you go first? | | Missing |  | 5.06 (0.0168) |  | 5.98 (0.0299) | 4.35 (0.0204) | <0.001 |
|  |  | Doctor or health care |  | 54.38 (0.0386) |  | 36.17 (0.0789) | 68.3 (0.0404) |  |
|  |  | Internet |  | 36.89 (0.0407) |  | 55.25 (0.0819) | 22.86 (0.0316) |  |
|  |  | Other |  | 3.67 (0.0112) |  | 2.59 (0.0116) | 4.49 (0.018) |  |
|  | |  |  |  |  |  |  |  |
| Overall, how confident are you that you could get advice or information about health or medical topics if you needed it? | | Missing |  | 2.27 (0.0083) |  | 1.1 (0.0073) | 3.15 (0.0138) | 0.8036 |
|  |  | Completely confident |  | 29.42 (0.0408) |  | 28.38 (0.0629) | 30.21 (0.0497) |  |
|  |  | Very confident |  | 37.38 (0.0395) |  | 38.95 (0.0738) | 36.18 (0.0458) |  |
|  |  | Somewhat confident |  | 26.58 (0.0327) |  | 25.52 (0.0615) | 27.38 (0.0384) |  |
|  |  | A little confident |  | 2.98 (0.0123) |  | 4.61 (0.0244) | 1.73 (0.0108) |  |
|  |  | Not confident at all |  | 1.38 (0.0053) |  | 1.44 (0.01) | 1.34 (0.006) |  |
|  | |  |  |  |  |  |  |  |
| In general, how much would you trust information about health or medical topics from each of the following? | A doctor | Missing |  | 1.45 (0.0062) |  | 0.98 (0.0072) | 1.81 (0.0096) | 0.5224 |
|  |  | A lot |  | 79.31 (0.0308) |  | 81.77 (0.0443) | 77.44 (0.0391) |  |
|  |  | Some – Not at all |  | 19.23 (0.0287) |  | 17.25 (0.0438) | 20.75 (0.037) |  |
|  | Family or friends | Missing |  | 5.14 (0.0152) |  | 2.35 (0.0162) | 7.28 (0.0233) | 0.8246 |
|  |  | A lot |  | 6.83 (0.0182) |  | 7.53 (0.0319) | 6.28 (0.0236) |  |
|  |  | Some – Not at all |  | 88.03 (0.0254) |  | 90.12 (0.0357) | 86.44 (0.0323) |  |
|  | Government health agencies | Missing |  | 5.05 (0.0129) |  | 0.98 (0.0072) | 8.16 (0.0224) | 0.5979 |
|  |  | A lot |  | 16.92 (0.0355) |  | 16 (0.0399) | 17.63 (0.0488) |  |
|  |  | Some – Not at all |  | 78.03 (0.0355) |  | 83.02 (0.0408) | 74.22 (0.0473) |  |
|  | Charitable organizations | Missing |  | 5.29 (0.0144) |  | 0.98 (0.0072) | 8.58 (0.0246) | 0.3039 |
|  |  | A lot |  | 0.95 (0.0049) |  | 1.55 (0.0098) | 0.49 (0.0042) |  |
|  |  | Some – Not at all |  | 93.76 (0.015) |  | 97.47 (0.0116) | 90.93 (0.0253) |  |
|  | Religious organizations and leaders | Missing |  | 4.32 (0.0116) |  | 0.98 (0.0072) | 6.87 (0.0201) | 0.5699 |
|  |  | A lot |  | 2.88 (0.0164) |  | 4.1 (0.0292) | 1.96 (0.0192) |  |
|  |  | Some – Not at all |  | 92.8 (0.0222) |  | 94.92 (0.0302) | 91.18 (0.0249) |  |
|  | |  |  |  |  |  |  |  |
| **Ownership and Technology Use** | | | | | | | | |
| Please indicate if you have each of the following. | | Missing |  | 1.56 (0.0083) |  | 1.51 (0.0154) | 1.6 (0.009) | 0.0454 |
|  |  | Tablet computer |  | 8.49 (0.0312) |  | 7.8 (0.0463) | 9.02 (0.0428) |  |
|  |  | Smartphone |  | 31.01 (0.0377) |  | 33.7 (0.057) | 28.96 (0.0464) |  |
|  |  | Basic cell phone only |  | 13.15 (0.0263) |  | 5.7 (0.0289) | 18.85 (0.0379) |  |
|  |  | None |  | 4.72 (0.012) |  | 1.07 (0.0082) | 7.51 (0.0202) |  |
|  |  | Multiple devices selected |  | 41.06 (0.0434) |  | 50.22 (0.0686) | 34.06 (0.0446) |  |
|  | |  |  |  |  |  |  |  |
| On your tablet or smartphone, do you have any apps related to health and wellness? | | Missing |  | 19.7 (0.0264) |  | 8.43 (0.0317) | 28.32 (0.0402) | 0.0975 |
|  |  | Yes |  | 38.34 (0.0378) |  | 53.98 (0.0737) | 26.39 (0.0448) |  |
|  |  | No |  | 30.17 (0.0349) |  | 30.19 (0.0625) | 30.16 (0.0402) |  |
|  |  | Don't know |  | 11.78 (0.0322) |  | 7.4 (0.0436) | 15.13 (0.0488) |  |
|  | |  |  |  |  |  |  |  |
| In the past 12 months, have you used the Internet to look for information about cancer for yourself? | | Missing |  | 23.78 (0.0363) |  | 11.46 (0.0406) | 33.2 (0.0524) | 0.8569 |
|  |  | Yes |  | 26.19 (0.0401) |  | 31.14 (0.0731) | 22.41 (0.0381) |  |
|  |  | No |  | 50.03 (0.0384) |  | 57.4 (0.0746) | 44.4 (0.0433) |  |
|  | |  |  |  |  |  |  |  |
| In the last 12 months, have you used the internet to watch a health-related video on YouTube? | | Missing |  | 0.9 (0.005) |  | 0 (0) | 1.59 (0.0088) | 0.0186 |
|  |  | Yes |  | 19.43 (0.0308) |  | 27.39 (0.0518) | 13.34 (0.0329) |  |
|  |  | No |  | 79.67 (0.0318) |  | 72.61 (0.0518) | 85.08 (0.0353) |  |
|  | |  |  |  |  |  |  |  |
| In the past 12 months, have you used a computer, smart phone, or other electronic device to look for health or medical information for yourself? | | Missing |  | 2.02 (.0085) |  | 1.77 (0.0168) | 2.15 (0.0086) | <0.001 |
|  |  | Yes |  | 68.21 (.0422) |  | 82.3 (0.0514) | 61.37 (0.0528) |  |
|  |  | No |  | 29.77 (.0424) |  | 15.93 (0.0518) | 36.48 (0.0507) |  |
|  | |  |  |  |  |  |  |  |

**References:**

1. Health Information national Trends Survey (H5-C3). 2019; <https://hints.cancer.gov/docs/Instruments/HINTS5C3_Annotated_Instrument_English.pdf>. Accessed January 29, 2021.
